# Linking Brassinosteroid and ABA Signaling in the Context of Stress Acclimation

**DOI:** 10.3390/ijms21145108

**Published:** 2020-07-20

**Authors:** Victor P. Bulgakov, Tatiana V. Avramenko

**Affiliations:** 1 Federal Scientific Center of the East Asia Terrestrial Biodiversity (Institute of Biology and Soil Science), Far Eastern Branch of the Russian Academy of Sciences, 159 Stoletija Str., Vladivostok 690022, Russia; avramenko.dvo@gmail.com; 2 Far Eastern Federal University, Sukhanova Str. 8, Vladivostok 690950, Russia

**Keywords:** ABA signaling, brassinosteroid signaling cascade, drought tolerance, priming, stress adaptation, stress memory

## Abstract

The important regulatory role of brassinosteroids (BRs) in the mechanisms of tolerance to multiple stresses is well known. Growing data indicate that the phenomenon of BR-mediated drought stress tolerance can be explained by the generation of stress memory (the process known as ‘priming’ or ‘acclimation’). In this review, we summarize the data on BR and abscisic acid (ABA) signaling to show the interconnection between the pathways in the stress memory acquisition. Starting from brassinosteroid receptors brassinosteroid insensitive 1 (BRI1) and receptor-like protein kinase BRI1-like 3 (BRL3) and propagating through BR-signaling kinases 1 and 3 (BSK1/3) → BRI1 suppressor 1 (BSU1) ―‖ brassinosteroid insensitive 2 (BIN2) pathway, BR and ABA signaling are linked through BIN2 kinase. Bioinformatics data suggest possible modules by which BRs can affect the memory to drought or cold stresses. These are the BIN2 → SNF1-related protein kinases (SnRK2s) → abscisic acid responsive elements-binding factor 2 (ABF2) module; BRI1-EMS-supressor 1 (BES1) or brassinazole-resistant 1 protein (BZR1)–TOPLESS (TPL)–histone deacetylase 19 (HDA19) repressor complexes, and the BZR1/BES1 → flowering locus C (FLC)/flowering time control protein FCA (FCA) pathway. Acclimation processes can be also regulated by BR signaling associated with stress reactions caused by an accumulation of misfolded proteins in the endoplasmic reticulum.

## 1. Introduction

In the last several years, there has been increased interest in the signaling system of brassinosteroids (BRs), and data has appeared on plant resistance to a lack of water upon activation of individual BR components [1,2]. The current model of BR signaling is that heterodimerization of protein brassinosteroid insensitive 1 (BRI1) and BRI1-associated receptor kinase (BAK1) initiates a signaling cascade that controls BR-responsive genes mainly through two homologous transcription factors, BRI1-EMS-supressor 1 (BES1) and brassinazole-resistant 1 protein (BZR1) [3]. The signal from the receptor is transmitted via brassinosteroid insensitive 2 (BIN2), a GSK3-like kinase taking the central place in BR signaling [4,5]. In the absence of BR, BIN2 is active and phosphorylates BZR1 and BES1, leading to loss of their DNA binding activity, exclusion from the nucleus by the 14-3-3 proteins, and degradation by the proteasome [3]. BR binding to the extracellular domain of BRI1 induces association and inter-activation between BRI1 and BAK1. Activated BRI1 then phosphorylates BSK1, which in turn dissociates from the receptor complex and interacts with BRI1 suppressor 1 (BSU1). BSU1 inactivates BIN2 by dephosphorylating its pTyr200, allowing the accumulation of unphosphorylated BZR1 and BES1. Dephosphorylated BZR1 and BES1 translocate to the nucleus and bind to their target genes to induce the BR response. Both BZR1 and BES1 bind to the BRRE (CGTGT/CG) and E-box (CANNTG) promoter elements through the conserved N-terminal DNA-binding domain and target a series of common genes to regulate BR-related responses [3,6,7,8,9,10,11,12,13,14,15,16]. 

However, this classic pathway leads to a decrease in growth, due to the relocation of resources in favor of protective reactions [2]. Fàbregas et al. [2] published an intriguing investigation to show that the *Arabidopsis thaliana* vascular brassinosteroid receptor BRL3 (receptor-like protein kinase BRI1-like 3) confers drought tolerance without decreasing growth. Authors observed that BRL3-overexpressing plants (BRL3ox) contained high levels of proline, sugars, and other osmoprotectants in non-stressed conditions and thus were better prepared for water deficiency due to a phenomenon known as priming [2]. The authors showed that drought resistance is under the control of cell-type specific BR signaling and that *BRL3* overexpression activates an alternative pathway of BR signaling. Analysis of BR signaling failed to provide a linear picture of the involvement of BRs in adaptation to drought stress [2]. As noted by the authors, overexpression of the canonical BRI1 pathway and its downregulation can both confer abiotic stress resistance. The phenotype of *Arabidopsis* BRL3ox plants demonstrates an active mechanism of drought tolerance driven by expression of the BRL3 receptor, but not the phenomenon known as drought avoidance (changes in stomatal conductance, leaf area, and leaf orientation).

BRL3 forms stable hetero-oligomers with BAK1, but not with BRI1, although BRL3 can complement BRI1 in different cell types and under different conditions [17]. The formation of distinct BR receptor complexes is interesting in itself, but it apparently does not explain the BRL3ox priming phenomenon. Analysis of integration with other signaling systems may be useful to unravel the mechanism of drought tolerance. In particular, *BRL3* overexpression caused an altered gene response of the ABA pathway.

ABA is a key phytohormone that regulates physiological and molecular responses to drought stress, including the accumulation of osmoprotectants [18]. Previous investigations of the BR signaling pathway showed a connection with ABA signaling (discussed below), with participation of other hormonal and light signaling systems [19,20]. Indeed, ABA signaling is closely related to abiotic stress resistance, and it can be assumed that ABA signaling interacts with the BRI1/BRL3 pathway. In brief, stress induces ABA accumulation and binding to its receptors of the PYL family to inhibit protein phosphatases 2C (PP2Cs). PP2C inactivation activates class 3 sucrose nonfermenting-1-related protein kinases (SnRK2s) that phosphorylate ABA-responsive element binding factors (ABFs). Activated ABFs initiate expression of responsive genes by binding to the *cis*-acting ABA response element (ABRE) [21]. 

Zhang et al. [22] noted: “Whether BR and ABA interaction is through modification or intersection of their signaling components or by independent or parallel pathways… remains a big mystery”. They found that ABA regulation of BR signaling depends on ABA signaling proteins, ABI1 and ABI2 [22]. The authors hypothesized that an activated BRI1 complex inhibits BIN2 kinase through an unknown mechanism and that ABA signaling is involved in BR signaling by regulating the GSK3-like kinase BIN2 or related proteins. Recently, Ren and colleagues showed how this happens (see section “Linking BRI1/BRL3 to the ABA signaling pathway”) [10]. In this review, we summarize data about links between BRI1 and BRL3 and the ABA signaling pathway at the level of protein–protein interactions. We propose new research trends in the study of the BR signaling pathway in relation to stress adaptation.

## 2. Linking BRI1/BRL3 to the ABA Signaling Pathway

**Figure 1:**
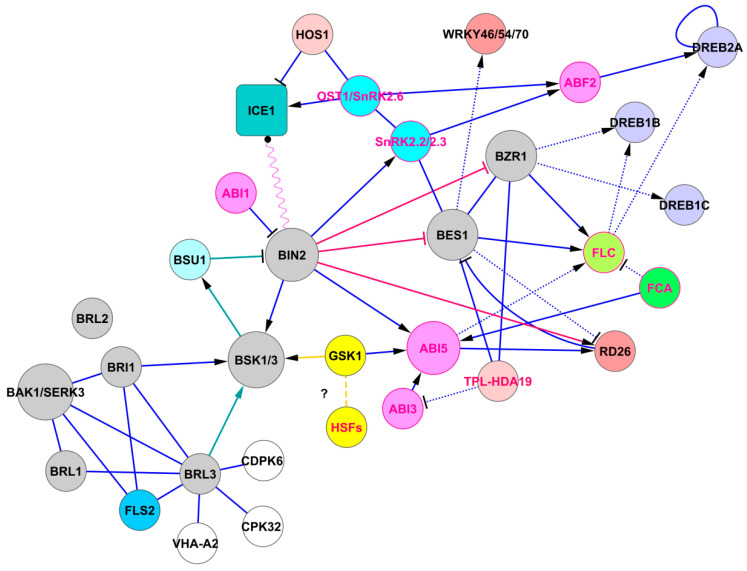
The pathway of BRL3 signaling. Brassinosteroid receptors BRL1, BRL3, and BRI1/BAK1 trigger the BR signaling pathway (proteins of the BR signaling system are shown in gray). BRL2 is not connected to this system. Solid lines represent protein–protein interactions presented in PAIR, IntAct, and BioGRID, and dashed lines represent possible interactions taken from STRING. Dotted lines represent transcriptional regulation. Green lines indicate signaling in the module BRL3 → BSK1/3 → BSU1 ―‖ BIN2. BIN2 regulates expression of BZR1 and BES1. BIN2 regulates drought tolerance directly by activating RD26, indirectly via BZR1-DREB1B and SnRK2.2/2.3-ABF2-DREB2A pathways. BIN2 also interacts with ICE1, implementing time-dependent regulation of the SnRK2.6/OST1-HOS1-ICE1 cold signaling module. Finally, BIN2 activates ABI5, an important concentrator of ABA signals. Red protein labels indicate that these proteins are involved in stress memory generation. These interactions were visualized using the program Cytoscape as described previously [27]. The data loaded into the program were obtained from PAIR version 3.3 [http://www.cls.zju.edu.cn/pair/]. The protein–protein interactions presented in PAIR were supplemented with data from BioGRID [http://thebiogrid.org/], UniProtKB [https://www.uniprot.org/], TAIR [https://www.arabidopsis.org/], IntAct [https://www.ebi.ac.uk/intact/interactors/], and STRING [https://string-db.org/] databases. Abbreviations: ABI1/3/5, ABA insensitive 1/3/5; ABF2, abscisic acid responsive elements-binding factor 2; BAK1, BRI1-associated receptor serine/threonine kinase; BES1, brassinazole-resistant 2; BIN2, brassinosteroid insensitive 2; BRI1, brassinosteroid insensitive 1; BRL1/2/3, serine/threonine-protein kinase BRI1-like 1/2/3; BSK1/3, BR-signaling kinases 1 and 3; BSU1, BRI1 suppressor 1; BZR1, brassinazole-resistant 1; CDPK6 and CPK32, calcium-dependent protein kinases; DREB1B,1C,2A, dehydration-responsive element-binding proteins; FCA, flowering time control protein; FLC, flowering locus C; FLS2, LRR receptor-like serine/threonine-protein kinase; GSK1, shaggy-related protein kinase iota; HDA19, histone deacetylase 19; HOS1, E3 ubiquitin-protein ligase HOS1; HSFs, heat shock factors; ICE1, inducer of *CBP* expression 1; RD26, NAC transcription factor; TPL, TOPLESS; SnRK2.2/2.3, SNF1-related protein kinases 2.2 and 2.3; VHA-A2, vacuolar proton ATPase.

There is still little data on the difference between BRI1 and BRL3 at the level of protein–protein interactions in the signaling cascade that regulates downstream reactions (Figure 1). Both BRI1 and BRL3 open the brassinosteroid signaling cascade by binding brassinolide [23] and might be linked to the ABA signaling system via the following pathway: BRI1/BRL3 → BR-signaling kinases 1 and 3 (BSK1/3) → BIN2 (Figure 1). However, BIN2 phosphorylates BSK1/3 [10,24,25], but not vice versa, and therefore we consider that the BRI1/BRL3 → BSK1/3 signaling module could not be related to the ABA signaling system. Instead, this module enters the branching signaling pathway related to plant immunity and development via somatic embryogenesis receptor kinases (SERKs) and the LRR receptor-like serine/threonine-protein kinase FLS2 (Figure 1; see also interactions in [26]).

Next, we focused on the study of BRL3-interacting partners and considered the possibility that proteins interacting with BRL3 perform a protective function. We manually checked all 45 BRL3-interacting proteins using BioGrid and TAIR annotations and found that almost all of them are signaling components related to plant immunity and development, with the exception of several proteins (https://thebiogrid.org/5872/summary/arabidopsis-thaliana/brl3.html). These include two calcium-dependent protein kinases (CDPK6 and CPK32), the vacuolar proton ATPase VHA-A2, and plasma membrane H^+^-ATPase 2 (AHA2). CDPK6 and CPK32 are involved in the ABA signaling pathway (BioGrid annotation), but their functionality in regards to BR signaling is unknown. Both VHA-A2 and AHA2 are important ATPases in establishing plant ion homeostasis under saline-alkali environmental conditions and act through the Salt-Overly-Sensitive signaling pathway and CBL-dependent calcium signaling [28,29,30]. Forty-sixth BRL3-interacting protein (not included in the BioGRID annotation) is a regulator of G-protein signaling 1 (RGS1) [31]. BRL3 phosphorylates RGS1 and thus functions in glucose sensing [31]. 

It is interesting to note that AHA2 also physically interacts with the serine/threonine-protein kinase BRI1-like 2 (BRL2). Paradoxically, the hub-type protein BRL2 (93 known interaction, BioGrid) is almost totally unrelated to BRL3, except for one common interaction, namely BRL3-AHA2. BRL2 interacts with numerous responsive proteins, including peroxidases, catalase CAT2, dehydrin ERD10, caffeic acid/5-hydroxyferulic acid O-methyltransferase (OMT1) and others, while BRL3 does not. These data are in accordance with the observation that BRL2, in contrast to BRI1 and BRL3, does not encode a functional BR receptor [23]. Summarizing the above information, we presume that the interaction of BRL3 with nearby proteins poorly explains BRL3-mediated drought tolerance. Thus, the unique effect of BRL3 on drought tolerance should be sought in long-distance signaling pathways.

A recent report by Ren et al. [10] indicates that BSK3 upregulates the serine/threonine-protein phosphatase BSU1 transcript and protein levels. Because BSU1 dephosphorylates and inactivates BIN2 [3,11], a signaling shunt, BSK1/3 → BSU1 ―‖ BIN2, may be established. The signaling module joining two subsystems is as follow: BRI1/BRL3 → BSK1/3 → BSU1 ―‖ BIN2 → BSK1/3 (Figure 1).

BSK3 physically interacts with BIN2 at the plasma membrane. In this interaction, BSK3 is a substrate of BIN2 kinase [10]. BSK3 phosphorylation by BIN2 allows the formation of BSK3/BSK1 heterodimer, BSK3/BSK3 homodimer, BSK3/BRI1 interaction, and BSK3/BSU1 interaction. If BIN2 is inhibited in this cascade, there will be consequences, since BIN2 blocks the activity of BZR1 and BES1 [3] and activates important components of the ABA signaling pathway (see below, section BIN2-based module). The BRL3 → BSK1/3 → BSU1 ―‖ BIN2 module can work independently of BRI1/BAK1 because BSK3 can activate BR signaling without a functional BRI1 receptor [10].

Previously, BSK3 had been described as a partially redundant regulator of brassinosteroid signaling [25] and now it is considered a scaffold protein to regulate overall BR signaling [10]. It is of interest as a participant in a “systemic foraging strategy” that increases the soil volume explored by the root system for the adaptation of plants to low nitrogen concentrations [32]. Therefore, BSKs could be central factors mediating the effects of the BRL3 receptor. BSKs join BRL3 to ABA signaling by modulating BIN2 activity because BIN2 interacts with central components of the ABA signaling pathway, such as the bZIP transcription factor ABI5 [33], protein phosphatase 2C ABI1 [34], with transcription factor ICE1 [35], and phosphorylates SNF1-related protein kinases SnRK2.2 and SnRK2.3 [36]. The interaction of BR signaling components with ABA signaling components can result in the generation of stress memory, i.e., the phenomenon described by Fàbregas et al. [2] as “acclimation”, in which ABA signaling components such as protein phosphatases 2C, ABI5, and SnRK2 kinases are involved in stress memory generation [27].

## 3. BIN2-Based Module

In the BR signaling pathway, BIN2 phosphorylates BES1 and BZR1 transcription factors to inhibit BR signaling through degradation of BES1 and BZR1 and by inhibiting their binding to DNA [12,13]. According to the conventional model of BR signaling, BRs act via BES1, which cooperates with WRKY46, WRKY54, and WRKY70, as well as other transcription factors, to activate plant growth-related genes and repress drought-responsive genes [14,15,16]. Under normal growth conditions, WRKY46/54/70 and BES1 positively regulate growth-related genes and negatively regulate the expression of drought-responsive genes. Under drought stress, WRKY46/54/70 and BES1 are destabilized which causes the repression of growth-related genes and activation of drought-related genes, which results in enhanced drought tolerance [15]. BES1 and the stress-responsive NAC transcription factor RD26 bind to a common promoter element, thus mutually inhibiting each other’s transcriptional activity ([14], see also Figure 1). The antagonistic interaction between BES1 and RD26 means that plant growth is reduced when plants are under water deficit, which induces RD26 to inhibit BR-induced growth, thus allowing the reallocation of resources to resist drought stress [14].

BIN2 positively regulates drought tolerance by upregulation of RD26 [34]. BIN2 directly interacts and phosphorylates RD26. In this way, we can see the involvement of ABA signaling components because protein phosphatase 2C ABI1 from the ABA pathway inhibits BIN2 kinase activity by dephosphorylation. The water deficit eliminates the ABI1-induced inhibition of BIN2 and further triggers drought tolerance by RD26. It should be noted that the expression of RD26 is also activated in BRL3-ox roots under a water deficit [2].

BIN2 negatively regulates the freezing tolerance, whereas BZR1 positively modulates the freezing tolerance [14,37]. BIN2 phosphorylates SnRK2.2 and SnRK2.3 (but not SnRK2.6/OST1), acting as a positive regulator of the ABA signaling pathway [36]. BZR1 acts via the CBF-dependent cold signaling pathway, directly activating *CBF1/DREB1B* and *CBF2/DREB1C* expression and by regulation of other cold-responsive (COR) genes [37]. Moreover, the freezing tolerance is regulated by the well-known SnRK2.6/OST1-HOS1-ICE1 signaling module that controls freezing tolerance via the CBF-dependent cold signaling pathway ([38], see also Figure 1). BIN2 interacts with SnRK2.6/OST1 but cannot phosphorylate it, suggesting that BIN2 acts through a non-conventional transphosphorylation site of SnRK2.6 [36]. BIN2 also interacts with ICE1, providing the attenuation of *CBF* expression (by time-dependent downregulation of ICE1 abundance) during the later stages of the cold stress response [14]. The silencing of BIN2 increases the resistance of plants to cold, while BIN2 overexpression results in hypersensitivity to freezing stress [37]. This effect was observed not only for acclimated but also for non-acclimated conditions [37].

The above information indicates that the signaling pathways passing through BIN2 lead to the regulation of both drought and cold protective reactions. This is in agreement with data from Fabregas et al. [2] regarding the enhanced expression of genes in BRL3ox compared to WT plants, in Gene Ontology (GO) categories, such as Response To Water Deprivation, Response to Temperature Stimulus, and Response To Cold or Cold Acclimation. As shown in Figure 1, BIN2 attenuation occurs in two ways, by the BR signaling component (BSU1) and the ABA signaling component (ABI1), which leads to both the weakening and strengthening of protective reactions to balance growth under stress conditions. The regulatory logic of this balance is not yet fully understood. To date, there is no data allowing discriminate functions of BRL3 and BRI1 in relation to the signaling chain BRL3 (or BRI1) → BSK1/3 → BSU1 ―‖ BIN2. Both receptors, BRI1 and BRL3 act through BIN2. We searched for other links between the BRI1 or BRL3 and BES1 or BZR in different databases and reports, and found no results, except for one mention in STRING, namely in the category “Co-Mentioned in PubMed Abstracts” (https://string-db.org/network/3702.AT4G39400.1). In earlier work, Kim et al. [3] suggested the existence of missing components in brassinosteroid signaling. It could be assumed that these missing components are numerous kinases with unknown functions that interact with BSK1/3. There are putative LRR receptor-like serine/threonine-protein kinases, AT1G51800 and AT5G10290, with an unknown function, as well as brassinosteroid-signaling kinases 5 and 8 and others, identified by Sreeramulu et al. [25] as BSK1/3-interacting proteins.

## 4. The Priming Phenomenon

Fàbregas et al. [2] hypothesized that the priming phenomenon might be a reason for drought tolerance and normal growth of BRL3-overexpressing plants. They based this assumption on the fact that the roots of BRL3ox plants are pre-loaded with osmoprotectant metabolites under normal conditions, and therefore they are better prepared for stress. Indeed, from a theoretical point of view, a plant can achieve this state (physiological equilibrium between growth and protection) by optimizing biochemical processes using memory generation processes. In higher plants, the stress memory phenomenon, known as ‘priming’ or ‘acclimation’, is achieved by chromatin modifications [39,40,41]. Describing the role of chromatin in water stress responses of plants, Han and Wagner [42] mentioned the role of histone modifications, histone (de)acetylases, histone lysine methyltransferases, histone arginine methyltransferases, histone variants, DNA methylation, and ATP-dependent chromatin remodeling complexes in memory generation. Most of these processes involve ABA signaling components [42].

Three types of stress-memory genes were described by Forestan et al. [43]: “transcriptional memory” genes, which have stable transcriptional changes persisting after recovery; “epigenetic memory candidate” genes, where stress-induced chromatin changes persist longer than the stimulus; and “delayed memory” genes, which are not immediately affected by the stress, but their expression patterns are perceived, stored, and later retrieved via chromatin remodeling for a delayed response.

**Figure 2:**
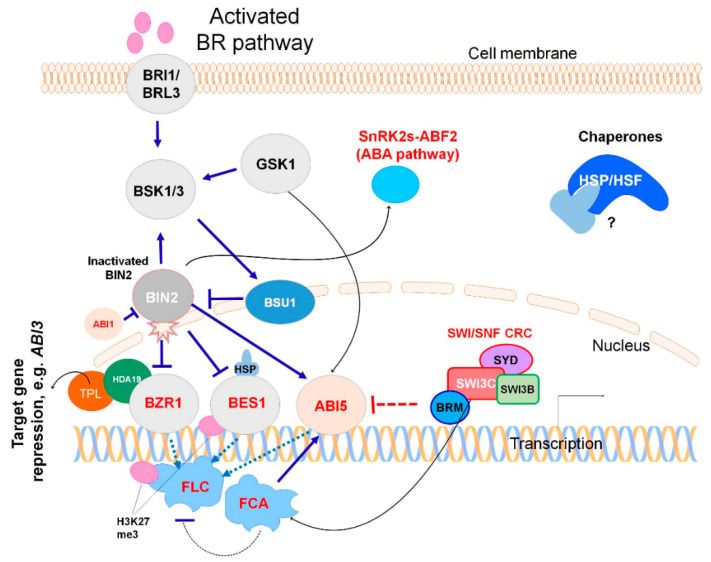
A model of stress memory generation by BR signaling. Solid lines represent protein–protein interactions and dotted lines represent transcriptional regulation. Proteins, involved in stress memory generation, are FLC and FCA (which substantially reduce plant water use and are important for heat and cold adaptation), TPL/HDA19 complex (ensures the epigenetic link between BR and ABA signaling through BZR1/BES1-ABI3-ABI5 interactions), and key components of the ABA signaling system such as SnRK2.2/2.3 and OST1/SnRK2.6, ABA-responsive element binding factors ABI3, ABI5 and ABF2 (involved in abiotic stress defense and stress memory). BZR1 recognizes and binds to a BRRE *cis* element in *FLC* and recruits H3K27 demethylase to dynamically modulate plant response to BR signals and environmental cues. SWI/SNF CRC is also a possible memory generator in this scheme. HSF function in BR signaling is possible, but has not been studied. Abbreviations: ABI5, ABA insensitive 5; BES1, brassinazole-resistant 2; BIN2, brassinosteroid insensitive 2; BRI1, brassinosteroid insensitive 1; BRL3, serine/threonine-protein kinase BRI1-like 3; BSK1/3, BR-signaling kinases 1 and 3; BRM, ATP-dependent helicase BRAHMA; BSU, BRI1 suppressor 1; BZR1, brassinazole-resistant 1; SWI/SNF CRC, (Switch/Sucrose non-fermenting, ATP-dependent chromatin remodeling complex); FCA, flowering time control protein; FLC, flowering locus C; GSK1, shaggy-related protein kinase iota; HDA19, histone deacetylase 19; HSP, heat shock protein; HSF, heat shock factor; SWI3B/3C, chromatin remodeling complex subunits; SYD, SWI2/SNF2-type ATPase; TPL, TOPLESS; SnRK2s, SNF1-related protein kinases 2.

The growing body of information indicates the involvement of BR signaling components in memory generation to stress. Shigeta et al. [44] suggested chromatin remodeling as a mechanism for the functioning of the BR pathway based on proteomic experiments. The authors proposed two mechanisms, specifically through the involvement of ATP-dependent chromatin remodeling complexes (CRC) or chromatin-modifying enzymes, such as histone deacetylases. Further, it was confirmed that histone modifying enzymes mediate the transcriptional activation of genes by components of the BR pathway [45,46]. Recently, Li et al. [47] showed that components of the BR pathway antagonize Polycomb silencing, thus introducing an epigenetic aspect in BR signaling. Possible mechanisms for generating memory in the BR signaling pathway are presented in Figure 2. The proteins involved in stress memory are marked in red.

Currently, there is no data to distinguish the specificity of the action of different BR receptors (BRI1, BRL1, and BRL3) at the level of protein–protein interactions. Therefore, the circuit shown in Figure 2 is applicable for the common BR pathway. The activated BR pathway leads to a state where BSU1 phosphatase inactivates BIN2, thus allowing activation of BZR and BES1 [10]. The regulator in ABA pathway, ABI1 phosphatase, can also dephosphorylate and destabilize BIN2 to inhibit BIN2 kinase activity [34]. Therefore, BIN2 functions as an important node in ABA-modulated BR signaling [22,34]. Activated BZR and BES1 in this pathway can in turn interact with stress-memory generating factors, such as TPL-HDA19, FLC/FCA and histone H3K27 demethylase (Figure 2). As components of the ABA pathway affect the BR pathway, the BR components also affect the ABA pathway. Specifically, ABI5 is regulated by BIN2 and GSK1, BIN2 regulates the function of SnRK2 kinases, and BZR1-TPL-HDA19 complex regulates the expression of *ABI3*. The DNA templates that carry response elements for binding factors are very different (ABRE, DRE, BRRE, and others), and they are not indicated in Figure 2. Note that the *cis-* and *trans*-regulatory logic of transcription factors involved is not considered, since it is not fully understood.

Since BRL3ox plants are more resistant to drought, they demonstrate a more stable rate of photosynthesis and transpiration during drought conditions, and have a larger preconditioned osmoprotective pool than WT plants [2]. It can be proposed that BRI1/BRL3 acts through memory factors that alter chromatin structure. It is not yet clear how the memory signal is passed, however it may be through the BRI1/BRL3 → BSK1/3 → BSU1 ―‖ BIN2 signaling pathway or others yet unknown. The BZR1/BES1 → TPL-HDA19 and BZR1/BES1 → FLC/FCA modules may be involved in such interactions. Below we consider possible options for these interactions.

BR-mediated repression of gene expression requires that histone deacetylases interact with TOPLESS (TPL) and that BZR1 associates with TPL and histone deacetylase HDA19 in vivo [45]. BZR1 recruits the TPL-HDA19 complex to BR-repressed promoters and mediates transcriptional repression via chromatin modification. The important role of BES1 is to create the BR-activated BES1-TPL-HDA19 repressor complex that controls epigenetic silencing of *ABI3* and *ABI5* [46]. This complex allows the suppression of ABA signaling during seedling development. Formation of a protein complex between BES1 or BZR1 and HDA19 is essential for regulation of drought stress tolerance [48].

The interaction of BR signaling components with FLC (MADS-box transcription factor encoded by flowering locus C) provides a new mechanism for drought resistance, where FLC substantially reduces plant water use [49]. FLC is also involved in long-term cold adaptation mediated by the epigenetic memory mechanism [50]. In the presence of BR, BZR1 and BES1-interacting MYC-like proteins (BIMs) bind to a BR-responsive element in the first intron of *FLC* and further recruits a histone 3 lysine 27 (H3K27) demethylase to suppress levels of the H3K27 trimethylation mark and thus antagonize Polycomb silencing at *FLC* [47]. FLC binds to numerous target genes to regulate their expression, including those involved in response to water deprivation, such as *CBF1/DREB1B* and *CBF3/DREB1A* [51]. The functioning of the *FLC* is regulated not only by BZR1 and BES1, but also by ABI5 [52], which establishes an additional connection between the ABA and BR pathways (Figure 2).

Another player in the BR-induced stress memory is the RNA-binding protein FCA, a component of flowering pathways in *Arabidopsis* and a regulator of *FLC* [53]. It has been shown previously that FCA interacts with SWI3A and SWI3B, components of the Switch/Sucrose non-fermenting, ATP-dependent chromatin remodeling complex (SWI/SNF CRC) [54]. FCA interacts with ABI5 and is essential for proper expression of ABI5-regulated genes involved in antioxidant defense and thermotolerance [55]. FCA not only regulates the function of many genes involved in adaptation to stress-induced ROS, heat, cold, and drought conditions via FLC and ABI5, but also adjusts the function of protective genes by itself, through chromatin modification and RNA metabolism [55]. Histone acetylation is important in the FCA-mediated thermal adaptation of developing seedlings, chlorophyll biosynthesis, and seedling photosynthetic fitness [56]. The FLC/FCA module functions not only in hot conditions, but also in cold, providing adaptation to winter conditions through an *FLC* antisense transcript *COOLAIR* [53,57]. This may explain why BRL3ox plants demonstrated high gene expression not only in the category “Response to Water Deprivation”, but also in the categories “Response to Temperature Stimulus” and “Cold Acclimation” [2]. It is interesting to note that these categories were also supplemented with GO category “Secondary Metabolic Process” [2]. Upregulation of genes related to secondary metabolism in BRL3ox plants can be explained by formation of the BR-activated BES1-TPL-HDA19 repressor complex, which acts via TPL on the jasmonate signaling system [58]. Additionally, activation might be mediated by FCA, which upregulates a number of secondary metabolism-specific biosynthetic genes and related transcription factors [55].

It is possible that the BR and ABA signaling pathways work simultaneously to ensure the priming effect on the drought tolerance of BRL3ox plants. In BRL3ox plants, ABA-dependent genes are upregulated, such as those that encode galactinol synthase 2 (GOLS2), dehydrin Xero 2 (LTI30), Em-like protein GEA1 (EM1), NAC transcription factor RD26, and cold and ABA inducible protein KIN1 [2]. Most of them are involved in the Response To Water Deprivation, Response To Cold or Cold Acclimation, and Response To Osmotic Stress (GO and BioGrid annotations). Of the ABA core signaling genes, protein phosphatases PP2C (ABI1, ABI2, and HAB1), transcription factors ABI3 and ABI5, SnRK2.2/2.3 kinases, and AREB/ABF transcription factors (such as ABF1, ABF2, ABF3, and ABF4) were shown to be involved in stress memory through interaction with SWI/SNF CRC [42,59]. The mechanism for memory generation through these interactions (Figure 2) may be realized via the ABA-chaperone pathway, where ABA-responsive elements (ABREs) recruit the SWI/SNF CRC to the chromatin template via ABFs and through the heat-shock transcription factors’ (HSFs) interaction with SWI/SNF CRC, histone-modifying enzymes, and other cofactors [27]. Indeed, the CRISPR/Cas9-mediated activation of AREB1/ABF2 through histone acetylation was shown to be useful for improving drought stress tolerance [60]. We must also consider that SWI/SNF chromatin remodelers interact with many players of the BR-ABA network, such as PP2Cs, SnRK2s, ABFs, BIM1, and others [27]. Han et al. [40] discovered that plants with mutated ATP-dependent helicase BRAHMA (BRM, a component of SWI/SNF CRC) acquired ABA hypersensitivity and increased drought resistance. BRM represses *ABI5* expression ([40], Figure 2). The authors suggested that the physiological role of BRM is to help plants avoid stress responses in the absence of stress. BRM is considered an important element in determining the allocation of resources between drought tolerance and growth [40].

## 5. Stabilization of Endoplasmic Proteins

Little is known about the connection between BR signaling and chaperones, which are necessary for stress adaptation. An important interaction occurs through BSK1/3 and GSK1, the shaggy-related protein kinase iota (synonyms: BIN2-LIKE 2, BIL2; Figure 1). We noted that the STRING database provides data indicating numerous interactions between *Arabidopsis* GSK1 and proteins known as heat-shock transcription factors (HSFs). The STRING data were accessed from the interaction of GSK and HSF homologs in non-plant organisms, such as *Saccharomyces cerevisiae*, *Caenorhabditis elegans,* and *Homo sapiens* (https://string-db.org/network/3702.AT1G06390.1). In many cases, these interactions were associated with stress reactions caused by an accumulation of misfolded proteins in the endoplasmic reticulum (ER).

These data prompted us to examine in more detail the literature about relationships between BR signaling and ER stress. In plants, there are no known interactions between GSKs and HSFs, but the association of BR signaling with ER stress signaling is well documented. A connection between ER stress signaling and BR-mediated growth and stress acclimation was shown by Che et al. [61]. They reported that *Arabidopsis* bZIP17 and bZIP28 transcription factors activate ER chaperone genes and BR signaling, which was required for stress acclimation and growth. Furthermore, Cui et al. [62] showed that UBC32, a stress-induced ubiquitin conjugation enzyme, connects the ER-associated protein degradation (ERAD) process, BR-mediated growth promotion, and salt stress tolerance [62]. BRI1 was also shown to be involved in this process.

The *Arabidopsis ethyl methanesulfonate-mutagenized brassinosteroid-insensitive 1 suppressor 7* (*EBS7*) gene, which encodes an ER membrane-localized ERAD component, is connected to the function of BRI1 and to stress tolerance via Hrd1a (ERAD-associated E3 ubiquitin-protein ligase Hrd1a), one of the central components of the *Arabidopsis* ERAD machinery [63]. Unlike in yeast and animal model systems, *Arabidopsis* ERAD components are just beginning to be studied, however recent investigations have revealed new important players and there is support for a connection between ER stress signaling and stress tolerance [64,65]. Our search in the databases showed that the interactome of eukaryotic organisms is enriched with numerous protein–protein interactions involving Hrd proteins, while there are no such interactions in the *Arabidopsis* interactome. In other words, *Arabidopsis* is underexplored in this regard. Summing up these data, we can hypothesize that BR signaling can increase stress resistance by stabilizing endoplasmic proteins. In the case of BRL3, it may be by the BRL3 (GSK1) → BSK1/3 pathway.

The chaperone signaling system comprises predominantly HSFs and heat-shock proteins (HSP), and peptidyl-prolyl cis-trans isomerases (PPIase), also called immunophilins [27]. Since the chaperone-type immunophilin FKBP42/TWD1 positively regulates the BRI1/BAK1 function and acts together with HSP90, it is possible that FKBP42/TWD1 and HSP90 assist the folding of membrane proteins [66]. Mutation in the *HvBRI1* gene causes a decreased HSP level and decreased *HSP* gene expression [67]. Although it is known that HSPs interact with the BR core components [68,69], there is no evidence of such interaction with HSFs. If it is established that BR signaling components interact with HSFs, then studies of BRs in terms of the implementation of stress memory will receive a new direction, since HSFs are the main sculptors of the epigenetic landscape [70].

## 6. Conclusions

In this review, we examined a key finding, recently reported by Fàbregas and colleagues [2], in which an increased expression of the BRL3 receptor provided resistance to a lack of water but did not impair plant development. Such cases, in relation to any stress, are quite rare, since resistance to any stressful condition is usually accompanied by growth retardation. Drought tolerance of plants is controlled by numerous signaling modules forming a branched network of protein–protein interactions. In this study, we examined all known interactions of the BR and ABA signaling pathways, but left out the signaling pathways of gibberellins and the light signaling system, which undoubtedly affect stress tolerance, but would greatly complicate the understanding of the described phenomenon.

In such cases as the one that was described by Fàbregas et al. [2], we should look for adaptation processes caused by memory generation. Ding et al. [39] postulated that under natural conditions, stress memory is activated by the previous dehydration stress, continues during the recovery period, and prepares the plant’s response to the next dehydration stress. This is surprising, but so far the BR pathway has not been extensively studied with respect to epigenetic changes and the generation of stress memory, and only the first steps have been taken on this path [45,46,47,68,71]. For the closest animal relatives of BRs, glucocorticoids, we observe an extensive field of research related specifically to changes in chromatin structure [72]. At present, it is still unclear whether BRs have a lesser effect on adaptive chromatin rearrangements or if they are simply underexplored in this regard.

We hypothesized that BRs may be involved in stress acclimation by three interconnected mechanisms. The first mechanism is that the signal passes through the module BRL3 (or BRI1) → BSK1/3 → BSU1 ―‖ BIN2 → BSK1/3, in which BIN2 is responsible for communication with ABA signaling and the BSK proteins serve as signal concentrators. The second mechanism is priming through chromatin modifications, in which BRL3 and other BR receptors could act collectively to ensure stress memory via BIN2 → SnRK2s → ABF2, BES1 or BZR1–TPL –HDA19 repressor complexes and BZR1/BES1 → FLC/FCA pathway. The third mechanism is stress acclimation by the BR-mediated stabilization of endoplasmic proteins in the ERAD process. New research prospects involving the BR signaling pathway in relation to stress adaptation are very intriguing and include the study of BR and ABA interaction pathways, chromatin modifications, and the ERAD process.

## Abbreviations

ABA	Abscisic acid
ABREs	ABA-responsive elements
BRs	Brassinosteroids
ER	Endoplasmic reticulum
ERAD	ER-associated protein degradation
GO	Gene Ontology
HSFs	Heat-shock factors
HSPs	Heat-shock proteins
PPIases	Peptidyl-prolyl cis-trans isomerases
SERK	Somatic embryogenesis receptor kinase
SWI/SNF CRC	Switch/Sucrose non-fermenting, ATP-dependent chromatin remodeling complex
